# Interpreting a Delayed Workup of Idiopathic Inflammatory Myopathy

**DOI:** 10.7759/cureus.55580

**Published:** 2024-03-05

**Authors:** Kristina Terrani, Ramzi Ibrahim, Sean P Ferris, Eric Brucks

**Affiliations:** 1 College of Medicine, University of Arizona College of Medicine – Tucson, Tucson, USA; 2 Department of Internal Medicine, University of Arizona College of Medicine – Tucson, Tucson, USA; 3 Department of Pathology, Division of Neuropathology, University of Michigan Medical School, Ann Arbor, USA

**Keywords:** neurological, immunological, weakness, inflammatory, myositis

## Abstract

Idiopathic inflammatory myopathies are a widely heterogeneous group of muscle diseases and encompass multiple clinicopathologic entities. Our case presentation describes a 70-year-old male who presented with progressively worsening dyspnea, along with worsening proximal muscle weakness in the bilateral lower extremities. Extensive clinical evaluation revealed a creatine kinase level of 105 IU/L, severe and chronic widespread myopathy seen on electromyography (EMG), and asymmetric but widespread muscle atrophy with fibro-fatty replacement seen on ultrasonography. Muscle biopsy specimen from the left deltoid was suboptimal but demonstrated characteristics that could be consistent with several clinicopathologic diagnoses, including sporadic inclusion body myositis (sIBM), immune-mediated necrotizing myositis (IMNM), antisynthetase syndrome (AS), and direct toxin-induced myopathy. Electron microscopy revealed tubulofilamentous inclusion associated with autophagic debris, finally rendering an accurate diagnosis. This case summary highlights the testing workflow required to diagnose a patient with an inflammatory myopathy and outlines the difficulty in establishing a diagnosis when the workup for an inflammatory myopathy is delayed and the muscle biopsy is suboptimal.

## Introduction

Idiopathic inflammatory myopathy (IIM) is a category of muscle diseases characterized by chronic, progressive, muscle weakness, and other related symptoms (depending on subcategory), with an overall incidence of 0.1-1 per 100,000 person-years and prevalence of 1.4-5.8 per 100,000 in the United States [1]. Causes include genetic factors, impairment in the embryological development process, alterations in the migration of progenitors, and environmental factors [2]. The major IIM subtypes include dermatomyositis, sporadic inclusion body myositis (sIBM), immune-mediated necrotizing myopathy (IMNM), and antisynthetase syndrome (AS) [3].

Polymyositis as a unique entity is currently a matter of debate in the neuromuscular field [3,4]. Although the various subtypes are similar in that they are inflammatory processes that involve muscle, they each have their own epidemiological ranges, clinical presentations, laboratory, and histological findings, as well as diagnostic criteria [1]. However, the main concerns for diagnosing the correct subtype of IIM are to be able to determine the best treatment and to communicate to patients the most correct prognosis. For example, while IMNM is often responsive to immunosuppressive therapy and has a high survival rate and good prognosis with treatment [5], sIBM is typically not responsive to immunosuppressive therapy and has a comparatively lower survival rate and poor prognosis, characterized by progressive functional disability [6].

Here, we present a patient who was seronegative for all myositis-specific and myositis-associated antibodies, with a suboptimal muscle biopsy showing results that could be consistent with several diagnoses. This case report adhered to the principles of the Helsinki Declaration through informed consent. 

## Case presentation

A 70-year-old Caucasian male presented to the emergency department for progressively worsening dyspnea in the past one month. The patient also noted an eight-year history of persistent weakness in his bilateral lower extremities. His past medical history was significant for hypertension, Graves' disease, type II diabetes mellitus, gastroesophageal reflux disease, and partial left lung lobectomy due to severe pneumonia. He denied any family medical history of autoimmune or rheumatologic diseases.

**Figure 1:**
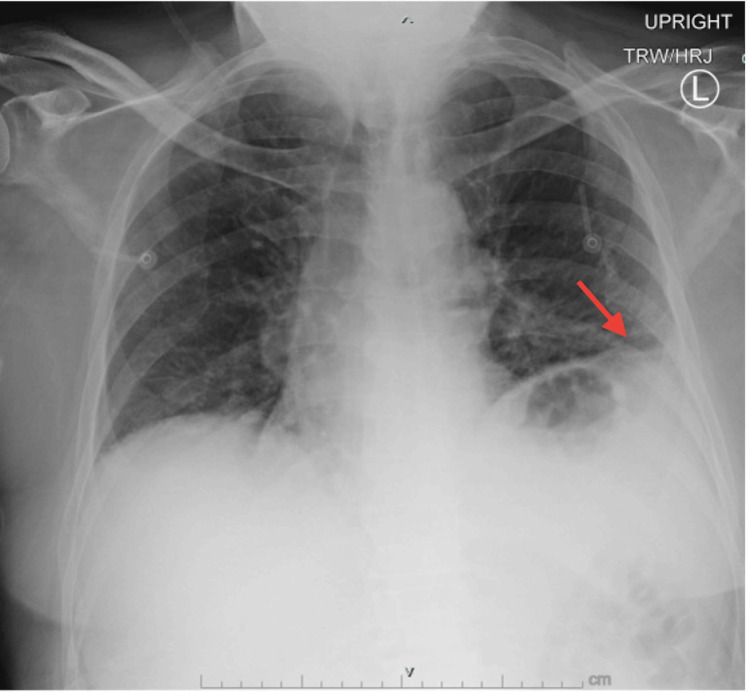
Chest radiograph showing low lung volumes and left basilar streaky opacity (red arrow) favoring atelectasis. No pleural effusions or pneumothorax are seen.

On presentation to the emergency department, vitals revealed blood pressure: 153/78 mmHg, heart rate: 95 beats per minute, and tachypneic: 27 respirations per minute on 7L high flow nasal cannula. The patient was afebrile and on examination, he had decreased breath sounds bilaterally and diminished respiratory effort, 4-/5 strength in bilateral upper extremities with the exception of left deltoid abduction which was 4+/5, 3/5 strength in bilateral lower extremities, and slowed bilateral patellar reflexes. Additional investigations were performed, and the results were as follows: mild leukocytosis at 11.2 K/uL, elevated hemoglobin at 18.1 g/dL, thrombocytopenia at 124K/uL, hypochloremia at 92 mmol/L, and hypercarbia at 42 mmol/L. Initial troponins were elevated at 48 ng/L (repeat 41 ng/L), C-reactive protein (CRP) level was 21.6 mg/L, and creatine kinase was 105 IU/L (within normal range). Initial venous blood gas revealed significant respiratory acidosis with a pH of 7.22, partial pressure of carbon dioxide (PCO2) of 123 mmHg, and bicarbonate level elevated to 51 mmol/L. The patient was initiated on bilevel positive airway pressure (BiPAP) with an improvement of venous blood gas to a pH of 7.28, PCO2 of 99 mmol/L, and bicarbonate of 47 mmol/L. Chest x-ray showed left basilar opacity favored to represent atelectasis (Figure 1). 

**Figure 2:**
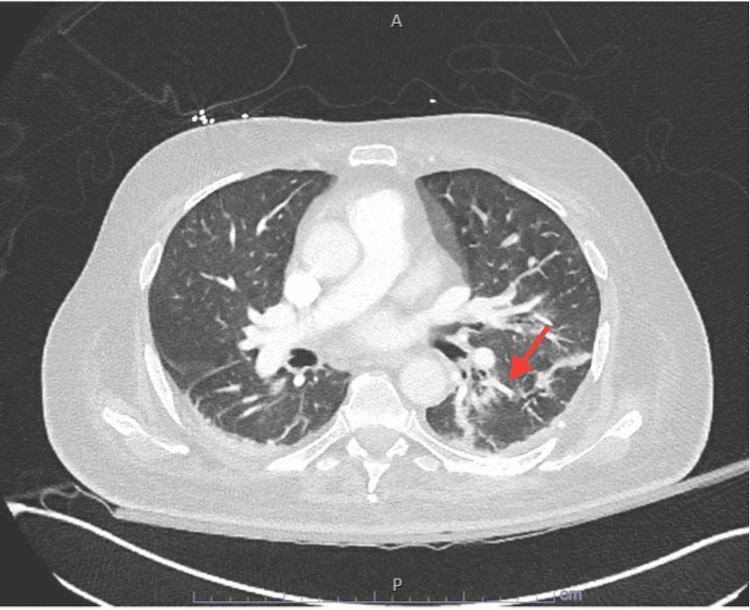
CTA of chest showing moderate streaky and consolidative opacities of bilateral lower lobes, with a minimal bilateral pleural effusion. No pulmonary embolus is seen. Red arrow shows streaky opacities seen in the left lung CTA: computed tomography angiogram

Computed tomography angiogram (CTA) of the chest revealed moderate bilateral lower lobar pneumonia and compression atelectasis in setting of poor ventilation (Figure 2).

The patient was started on ceftriaxone 1 gram daily and azithromycin 500 mg daily to cover for community-acquired pneumonia, and due to his acute-on-chronic respiratory failure and BIPAP requirements, the patient was admitted to the medical intensive care unit (MICU).

After acute stabilization in the MICU and admission to the medicine floor, additional tests and procedures were performed to investigate the causes of his proximal muscle weakness and respiratory failure. The patient provided us with a more detailed history and stated that he had been evaluated by a pulmonologist for his respiratory issues a month before presentation, and per the pulmonologist’s evaluation, there was no evidence of neuromuscular respiratory weakness. Out-of-system records were obtained which revealed a decreased forced vital capacity (FVC) consistent with a history of severe pneumonia. His minimal inspiratory pressure (MIP)/minimal expiratory pressure (MEP) was normal at that time; however, his diffusing capacity for carbon monoxide (D_LCO_) was reduced at 56%.

Animal and environmental allergen tests were all negative, as well as autoantibody tests (PL-7, PL-12, Mi22, Ku, EJ, OJ, SRP, Jo-1), IgA, IgG, and IgM. IgE levels were elevated at 295 and CRP levels were elevated at 10.2. Due to the discrepancies in his pulmonary function tests and history, and history of intermittent episodes of muscle weakness for about eight years which worsened in the last six months, the pulmonologist referred him to a neurologist to be evaluated for this muscle weakness. An electromyogram (EMG) was performed, which showed electrodiagnostic evidence of severe, chronic widespread myopathy (Table 1), as well as an ultrasound of the vastus lateralis which demonstrated asymmetric but fairly widespread muscle atrophy with fibro-fatty replacement involving essentially all muscles tested.

**Table 1:** Electromyography results showing that with each activation, all muscles had small amplitude, short duration motor unit potentials firing with early recruitment, stable polyphasic units with multiple thin spikes. FDP: flexor digitorum profundus; FDS: flexor digitorum superficialis; PPP: polyphasic potential; R: right

Muscle	Amplitude	Duration	PPP	Stable	Recruitment
R. Tibialis Anterior	Low	Short	Few	Yes	Early
R. FDP	Low	Short	Frequent	Yes	Early
R. FDS	Low	Short	Frequent	Yes	Early
R. Vastus Medialis	Low	Short	Few	Yes	Early
R. Triceps Brachii	Low	Short	Normal	Normal	Early
R. Biceps Brachii	Low	Short	Few	Yes	Early
R. Deltoid	Low	Short	Few	Yes	Early

Physical exam performed by the neurologist revealed a slightly abnormal casual gait with a tendency to lock knees, with normal neck flexor and extensor strength. Muscle bulk was normal in all four limbs, with no atrophy or fasciculations present. The formal muscle strength examination is detailed in Table 2.

**Table 2:** Neurological strength evaluation with the formal muscle strength examination. Graded on the Medical Research Council (MRC) Scale for Muscle Strength, where 5/5 is normal.

Muscle	Right	Left
Upper extremities		
Deltoid	4	4
Biceps	3+	4-
Triceps	3+	4-
Wrist extensor	4	5
Wrist flexor	4	4
Flexor digitorum superficialis	5	5
Flexor digitorum profundus	4-	4-
Finger extensors	4+	4+
Lower extremities		
Hip flexors	4	4
Quadriceps	4	4
Hamstrings	4	4
Foot dorsiflexors	4-	4+
Foot plantarflexors	4+	4+

During his hospitalization, surgery was consulted to perform a biopsy of the left deltoid, which was the most relatively preserved muscle per his ultrasound results. The patient had not received any pre-biopsy steroid treatment. After the patient was stabilized and back to his baseline oxygen requirements, he was discharged, and the rest of the investigation was performed in the outpatient setting.

**Figure 3:**
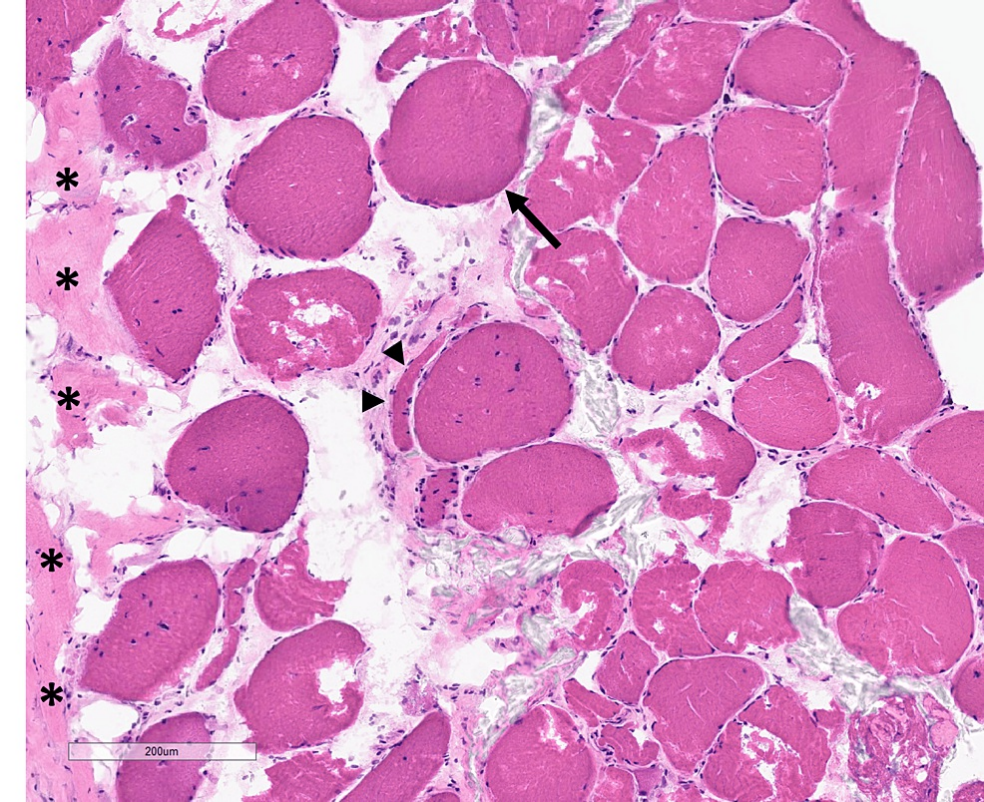
H&E staining of frozen muscle tissue showing variation in muscle fiber diameters: scattered atrophic fibers (arrowheads) and hypertrophic fibers (arrow); muscle fiber diameters range from 15 to 150 microns, and increased internal nuclei in fibers directly adjacent to tendinous insertion site (left side, asterisks).

Muscle biopsy produced two tissue fragments (one formalin-fixed paraffin-embedded (FFPE) specimen, and one fresh-frozen fragment), both showing myotendinous junction surrounded by variable amounts of muscle fibers. There was extensive and severe fatty replacement, scattered atrophic fibers, scattered pyknotic nuclear aggregates, and numerous hypertrophic fibers with diameters up to 150 microns (Figure 3).

**Figure 4:**
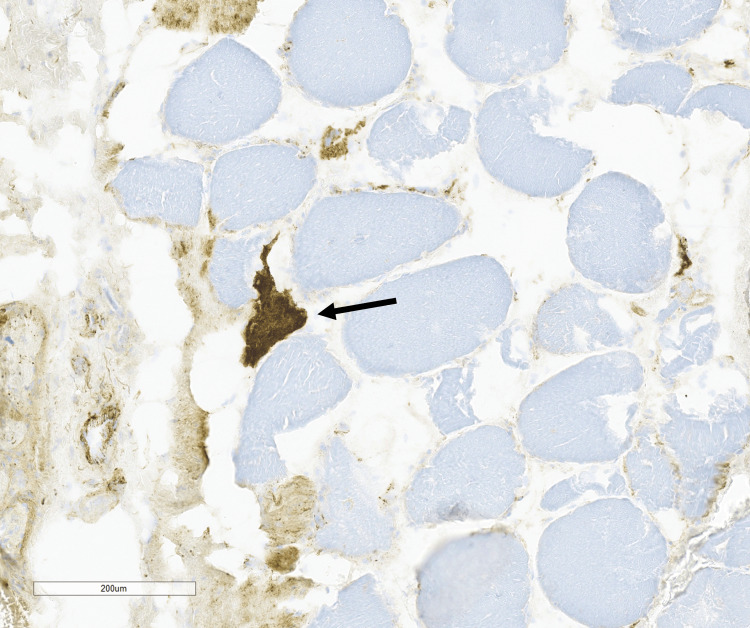
C5b9 stain highlights a rare necrotic fiber (arrow).

Only a rare necrotic fiber was seen on the H&E stain and highlighted by the C5b9 stain (Figure 4), and a single regenerating fiber was seen on the alkaline phosphatase stain. 

**Figure 5:**
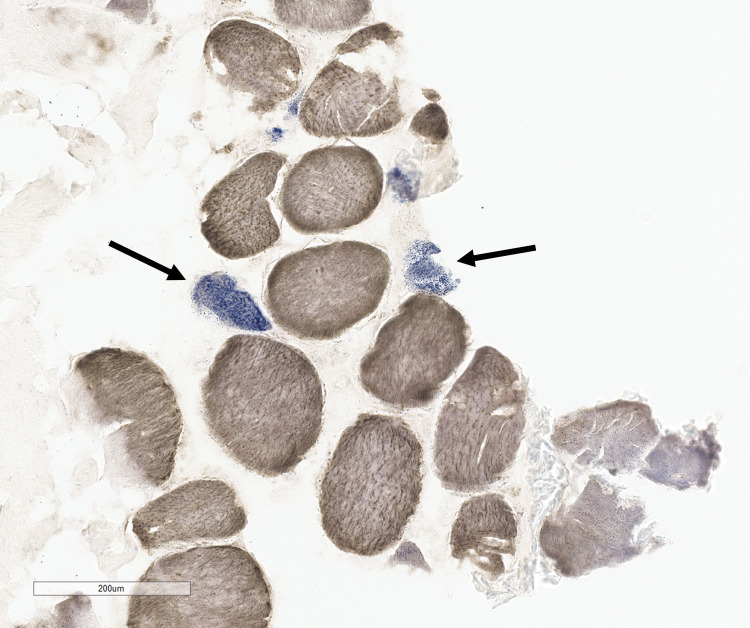
COX-SDH stain showing increased COX-negative fibers (blue stain, arrows); 7-8% of all fibers.

There were only approximately 200-250 total fibers in the fresh-frozen tissue section, and cytochrome oxidase (COX)-negative fibers were increased (approximately 7-8% of all fibers COX-negative (Figure 5)). 

**Figure 6:**
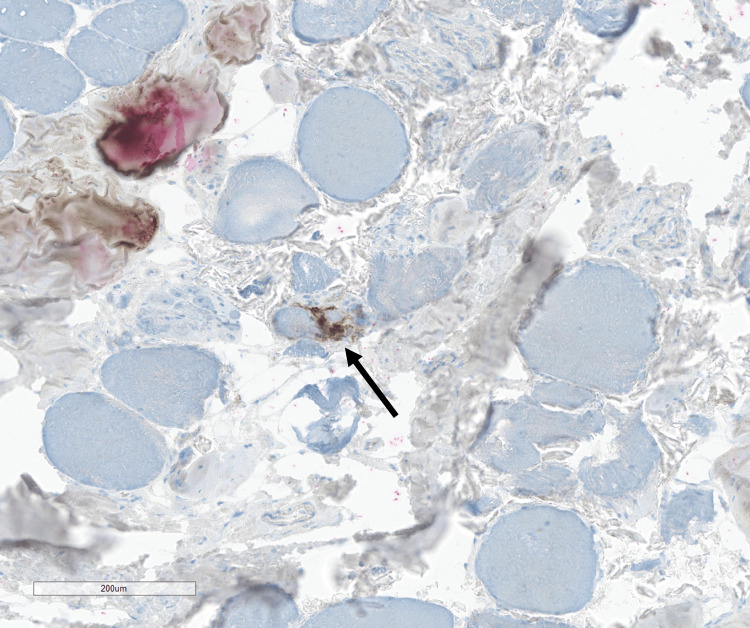
CD3/CD20 stain demonstrating a single small focus of endomysial CD3-positive T cells (brown, arrow) without any significant population of CD20-positive B cells (red).

There was only a single small focus of endomysial T-cell predominant lymphocytic inflammation (highlighted by CD3/20 co-stain (Figure 6). 

**Figure 7:**
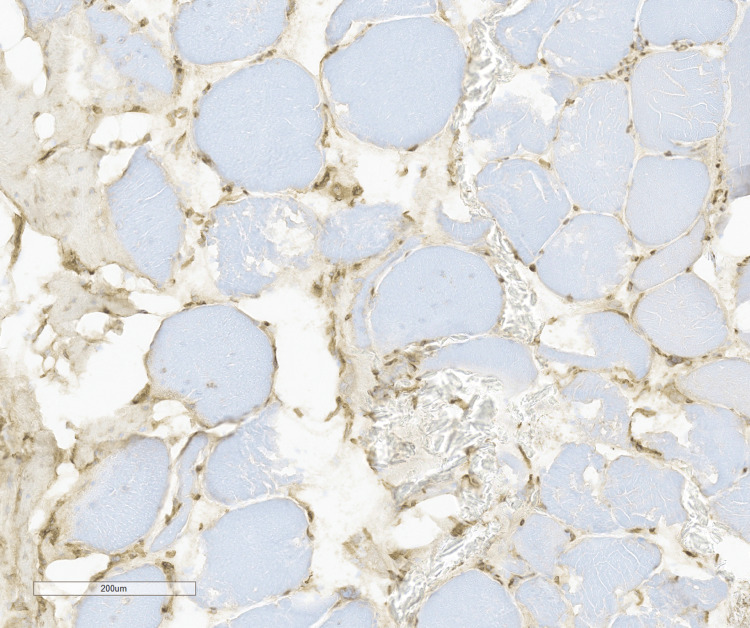
MHC-I stain is negative for sarcolemmal staining in the muscle fibers.

MHC-I stain was negative for significant sarcolemma staining in muscle fibers (Figure 7). 

p62 stain showed scattered fibers with sarcoplasmic punctate/granular staining but was negative for abnormal coarse protein aggregates. TDP-43 stain was entirely negative. Rimmed vacuoles were not identified on trichrome or H&E stains. Scattered atrophic fibers were esterase-positive, but there was no definite evidence of chronic neurogenic rearrangement. A pathologic diagnosis of "features of chronic myopathy" was rendered.

Two months after admission, the neurologist ordered additional tests: serum anti-5'-nucleotidase cytosolic 1a (NT5C1a) antibody, anti-signal recognition particle (SRP) antibody, and anti-3‑hydroxy-3-methylglutaryl-CoA reductase (HGMCR) antibody tests which were all negative, and aldolase levels which were within normal limits. The neurologist’s review of physical exam findings showing profound humoral, deep finger flexor, and quadriceps weakness along with EMG and ultrasound results, seronegative antibody panels, and inconclusive muscle biopsy findings led to strong clinical suspicion for sIBM. Immune-mediated necrotizing myopathy (IMNM) was ruled out clinically, as the patient’s weakness had been present for at least 15 years with an insidious onset and gradual progression. At this point, no immunosuppressive treatment was given.

**Figure 8:**
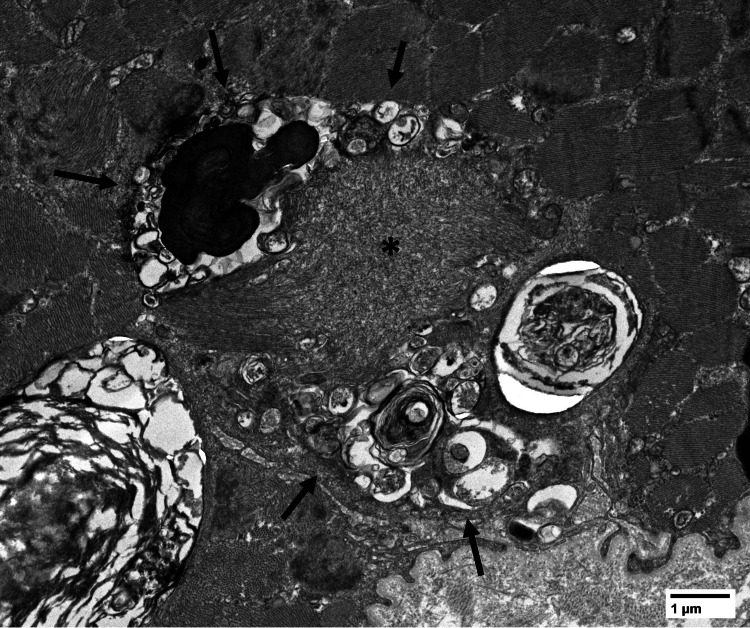
Electron microscopy revealing tubulofilamentous inclusion (asterisk) associated with autophagic debris and membrane-like whorls (arrows).

Due to the strong clinical suspicion for sIBM, electron microscopic (EM) analysis was performed on the muscle biopsy material. Epon-embedded toluidine blue-stained semithin sections demonstrated a rare fiber with an amorphous sarcoplasmic inclusion, and a rare fiber with a possible sarcoplasmic autophagic vacuole. EM analysis demonstrated at least one tubulofilamentous inclusion associated with autophagic debris and membrane-like whorls (Figure 8). 

Mitochondria appeared to be relatively normal in number, but there were scattered mitochondria with abnormal concentric cristae and/or punctate dense granules. No crystalline or paracrystalline arrays were identified. There was minimal focal disorganization of the myofibrillary apparatus. Lipids were within normal limits, and there was no significant increase in subsarcolemmal glycogen. After this additional finding of tubulofilamentous inclusions by EM analysis, a final integrated pathologic diagnosis of "features of chronic myopathy compatible with inclusion body myositis/myopathy" was rendered.

## Discussion

IIM is a group of diseases characterized by chronic, progressive, proximal muscle weakness [7]. The patient in this case presented with an unknown subclass of IIM, and a muscle biopsy was obtained late in the disease course. The patient had physical exam and ultrasound evidence of widespread muscle atrophy and fatty replacement, and the muscle sample (biopsied from the patient’s most relatively preserved muscle) was still suboptimal because there were a relatively small number of muscle fibers present, and the majority of muscle fibers were adjacent to a collagenous myotendinous insertion site. Fibers adjacent to myotendinous insertion sites often demonstrate “pseudo-myopathic” features including multiple internal nuclei and fiber splitting [8]. Hypertrophic fibers were present, and of note, hypertrophic fibers have been reported to be frequent in patients with sIBM, but rare in patients with polymyositis [9]. Therefore, it was difficult to definitively determine whether some changes were due to proximity to the collagenous tendinous insertion site, or true myopathic changes. Multiple immunohistochemical and/or enzyme histochemical stains were performed (MHC-I, C5b-9, COX/succinic acid dehydrogenase (SDH), CD3/CD20, and p62) in an attempt to come to a more specific diagnosis, and the significance of those results are discussed below.

Major histocompatibility complex Class I (MHC-I) immunohistochemical (IHC) staining is in general a sensitive marker for the diagnosis of inflammatory myopathy but can be focal or even negative in some subclasses (for instance, anti-MDA5 positive dermatomyositis) [10]. While most reports indicate that MHC-I is upregulated in essentially all cases of sIBM [11], at least one study reported that MHC-I was only increased in fibers involved by inflammation in patients with sIBM [12]. Therefore, the significance of the negative MHC-I result for this patient is not entirely clear but may be secondary to variable MHC-I staining within the patient’s muscles, and could correlate with the almost complete lack of lymphocytic inflammation in the biopsy material. MHC-II staining is less commonly used as an investigative stain for inflammatory myopathies, and one study indicated that it has a high specificity for sIBM [13]. However, as MHC-II staining is not available at the performing institution, MHC-II staining was not performed in this case.

Terminal complement complex/membrane attack complex (C5b-9) IHC staining is a useful stain for the evaluation of muscle biopsy samples, and most notably highlights complement deposition on perifascicular capillaries in many cases of dermatomyositis [14]. Capillary C5b-9 staining has also been reported in muscle biopsies from patients with diabetic peripheral neuropathy, and other inflammatory myopathies [15]. Sarcolemmal C5b9 staining on muscle fibers is also a useful diagnostic pattern and can be seen in IMNM and some other inflammatory myopathies [10]. Neither of these staining patterns was seen in this patient’s muscle biopsy, but C5b-9 staining can also highlight necrotic fibers and was useful in this patient’s case to confirm the presence of a rare necrotic fiber.

COX enzyme histochemical staining is used in histological analysis of muscle biopsies to detect fibers with mitochondrial abnormalities. Co-staining for succinyl dehydrogenase (SDH) with a blue chromagen facilitates the detection of fibers with absent COX activity (COX-negative fibers have retained SDH staining and appear blue). Increased COX-negative fibers can be seen in mitochondrial myopathies, sIBM, so-called “polymyositis with increased COX-negative fibers”, and in association with aging [11]. The current patient’s muscle biopsy showed approximately 7-8% COX-negative fibers, which is increased beyond what might be expected for a normal muscle from a 70-year-old male.

CD3/CD20 immunohistochemical stain highlights T-cells and B-cells, respectively. Endomysial, perivascular, and/or perimysial inflammation is a core histologic feature of many IIMs; however, the level of inflammation in biopsies may be decreased by pre-biopsy steroid treatment (although there was no pre-biopsy steroid treatment in this patient’s case) [10]. Muscle biopsies from most patients with sIBM show a significant inflammatory component, whereas most cases of IMNM show a “pauci-immune” histologic appearance [10,11]. The significance of only a rare focus of endomysial T cell inflammation in the muscle biopsy from our patient is not entirely clear but appears to correlate with the lack of MHC-I staining.

The initial clinical findings and muscle biopsy result of “features of chronic myopathy” suggested a differential diagnosis including IMNM, sIBM, AS, and toxin-induced myopathy. Toxin-induced myopathy is essentially excluded clinically considering the slow progress of the patient’s weakness. IMNM clinically presents between ages 40 and 60 years with proximal symmetric muscle weakness and occasionally some cutaneous findings. It can be associated with pulmonary disease, arthritis, statin use, and gastrointestinal symptoms [16]. Laboratory results reveal elevated serum creatine kinase and CRP. Anti-HMGCR or anti-SRP antibodies are positive in approximately one-third of IMNM patients, respectively; however, somewhere between 20-40% of patients are negative for these autoantibodies (seronegative IMNM) [16]. Muscle biopsies typically show prominent myonecrosis with a relative lack of lymphocytic inflammation. MHC-I staining can be weak, focal, or absent, but is usually not strong and diffuse [16]. A fine punctate/granular sarcoplasmic p62 staining pattern in a significant amount of scattered fibers may be fairly specific for IMNM, but this pattern was not well-established in this patient’s case, and non-specific p62 staining is also a common finding in IIM muscle biopsy specimens.

AS presents clinically as an inflammatory myopathy with proximal, symmetric muscle weakness, and extramuscular findings such as interstitial lung disease, Raynaud’s phenomenon, and arthritis [17]. According to both sets of proposed classification criteria for AS, there must be serum auto-antibodies directed against an aminoacyl tRNA synthetase, i.e. anti-Jo1 (histidyl), anti-PL7 (threonyl), and anti-Pl12 (alanyl), amongst others, as well as one of the above clinical symptoms [18]. While our patient did meet the criteria for clinical symptomatology, he was seronegative for all AS-specific antibodies. Muscle biopsies from patients with AS-associated myositis demonstrate a classical histological finding of perifascicular myofiber necrosis, and this finding was not present in our patient’s limited biopsy. Of note, it is important to consider this diagnosis in patients with unexplained acute or chronic interstitial pneumonia, as our patient did. Overall, prognostic outcomes are primarily determined by the severity of the interstitial lung disease in these patients. Other less common prognostic factors include older age, male gender, African American ethnicity, symptomatic, and steroid-resistant interstitial lung disease, as well as low initial FVC/D_LCO_ numbers [19].

sIBM generally affects older individuals, more commonly males, and has a more insidious onset and slower progression than the other inflammatory myopathies, as our patient did. However, the distribution of weakness tends to be more distal in the upper extremities and less symmetric, which was not the case in our patient. EMG in patients with sIBM will show polyphasic motor unit action potentials of long duration and large amplitude, due to the chronicity of the disease (as opposed to the actual neurogenic degenerative process) [20], which is not consistent with our patient’s results. In addition, our patient was seronegative for antibodies against cytosolic 5'-nucleotidase 1A (NT5C1A), but it is not present in approximately half of patients. Muscle biopsies from patients with sIBM typically show prominent endomysial lymphocytic inflammation, muscle fibers with rimmed vacuoles, and diffuse MHC-I positivity, none of which were present in this patient’s muscle biopsy [11]. However, MHC-I expression can reportedly be varied or focal in a subset of cases of sIBM [21]; therefore, in retrospect, the lack of significant sarcolemmal MHC-I staining in muscle fibers in this patient’s muscle biopsy does not entirely exclude the possibility of sIBM. Muscle fiber inclusions can highlighted by Congo Red stain in some cases [22], and electron microscopy can demonstrate intranuclear and/or cytoplasmic tubulofilamentous inclusions, autophagic/rimmed vacuoles, and mitochondrial abnormalities [22].

For our patient, further EM analysis demonstrated a new finding of a tubulofilamentous inclusion body associated with autophagic debris. This finding is considered by some to be a pathognomonic feature of inclusion body myositis [22]. The increased COX-negative fibers in this patient’s biopsy material are also compatible with sIBM. Therefore, although a number of pathologic features of sIBM were not identified in this patient’s muscle biopsy, the constellation of clinicopathological findings in this case is most supportive of sIBM. 

## Conclusions

Although muscle biopsy is the gold standard for diagnosis of IIM, in cases with late/delayed workup, muscle biopsy results can be inconclusive for various reasons (in this case extensive muscle atrophy contributed to suboptimal biopsy material). This ultimately has an effect on the prognostic abilities of physicians. Therefore, it is imperative for physicians to recognize the symptoms of myopathies to allow for early diagnosis and prompt workup.
